# The future of medical diagnostics: review paper

**DOI:** 10.1186/1758-3284-3-38

**Published:** 2011-08-23

**Authors:** Waseem K Jerjes, Tahwinder Upile, Brian J Wong, Christian S Betz, Henricus J Sterenborg, Max J Witjes, Kristian Berg, Robert van Veen, Merrill A Biel, Adel K El-Naggar, Charles A Mosse, Malini Olivo, Rebecca Richards-Kortum, Dominic J Robinson, Jennifer Rosen, Arjun G Yodh, Catherine Kendall, Justus F Ilgner, Arjen Amelink, Vanderlei Bagnato, Hugh Barr, Lina Bolotine, Irving Bigio, Zhongping Chen, Lin-Ping Choo-Smith, Anil K D'Cruz, Ann Gillenwater, Andreas Leunig, Alexander J MacRobert, Gordon McKenzie, Ann Sandison, Khee C Soo, Herbert Stepp, Nicholas Stone, Katarina Svanberg, I Bing Tan, Brian C Wilson, Herbert Wolfsen, Colin Hopper

**Affiliations:** 1 The "Head and Neck Optical Diagnostics Society" Council, The International Society of Minimally Invasive Diagnostics, University College London, London, UK; 2 Department of Trauma and Orthopaedic Surgery, Leeds Teaching Hospitals NHS Trust, Leeds, UK; 3 Academic Department of Trauma and Orthopaedic Surgery, School of Medicine, University of Leeds, Leeds, UK; 4 UCL Department of Surgery, Division of Surgery and Interventional Science, London, UK; 5 Department of Otolaryngology/Head and Neck Surgery, Barnet and Chase Farm Hospitals NHS Trust, London, UK; 6 UCLH Head and Neck Centre, University College London Hospitals, London, UK; 7 The Beckman Laser Institute and Medical Clinic, The University of California Irvine, Irvine, CA, USA; 8 Department of Otorhinolaryngology, Head & Neck Surgery, Ludwig Maximilian University, Munich, Germany; 9 Center for Optical Diagnostics and Therapy, Erasmus University Medical Center, Rotterdam, the Netherlands; 10 Department of Oral & Maxillofacial Surgery, University Medical Center Groningen, Groningen, the Netherlands; 11 Dept. of Radiation Biology, The Norwegian Radium Hospital, Montebello, Norway; 12 Virginia Piper Cancer Institute, Abbott Northwestern Hospital, Minnesota, USA; 13 Department of Pathology, The University of Texas M.D. Anderson Cancer Center, Houston, Texas, USA; 14 National Medical Laser Centre, University College London Department of Surgery, London, UK; 15 School of Physics, National University of Ireland, Galway, Ireland; 16 Department of Bioengineering, Rice University, Houston, USA; 17 Department of Radiation Oncology, Erasmus University Medical Center, Rotterdam, the Netherlands; 18 Department of Surgery, Boston University School of Medicine, Boston, USA; 19 Physics and Astronomy Department, University of Pennsylvania, Philadelphia, USA; 20 Biophotonics Research Group, Gloucestershire Hospitals NHS Foundation Trust, Gloucester, UK; 21 Department of Otorhinolaryngology, Plastic Head and Neck Surgery, Aachen University Hospital, RWTH Aachen, Germany; 22 Centro de Pesquisa em Óptica e Fotônica, University of Sao Paulo, Sao Carlos, SP, Brazil; 23 Research Centre for Automatic Control (CRAN), Nancy-University, UMR CNRS, France; 24 Department of Biomedical Engineering, Electrical & Computer Engineering, Physics, Boston University, Boston, USA; 25 Department of Biomedical Engineering, Beckman Laser Institute, University of California, Irvine, USA; 26 National Research Council Canada-Institute for Biodiagnostics, Winnipeg, Manitoba, Canada; 27 Department of Oral & Maxillofacial Surgery, Tata Memorial Hospital, Mumbai, India; 28 Department of Head and Neck Surgery, Division of Surgery, The University of Texas M. D. Anderson Cancer Center, Houston, TX, USA; 29 Michelson Diagnostics, Orpington, Kent, UK; 30 Department of Histopathology, Imperial College and The Hammersmith Hospitals, London, UK; 31 National Cancer Centre, Singapore, Singapore; 32 LIFE Center, University Clinic Munich, Munich, Germany; 33 Division of Oncology, Lund University Hospital, Lund, Sweden; 34 Department of Head & Neck Oncology & Surgery, The Netherlands Cancer Institute - Antoni van Leeuwenhoek Hospital, Amsterdam, the Netherlands; 35 Division of BioPhysics and BioImaging, Ontario Cancer Institute, Ontario, Canada; 36 Department of Medical Biophysics, Faculty of Medicine, University of Toronto, Toronto, Canada; 37 Division of Gastroenterology and Hepatology, Mayo Clinic, Florida, USA

## Abstract

While histopathology of excised tissue remains the gold standard for diagnosis, several new, *non-invasive *diagnostic techniques are being developed. They rely on physical and biochemical changes that precede and mirror malignant change within tissue. The basic principle involves simple optical techniques of tissue interrogation. Their accuracy, expressed as sensitivity and specificity, are reported in a number of studies suggests that they have a potential for cost effective, real-time, *in situ *diagnosis.

We review the Third Scientific Meeting of the Head and Neck Optical Diagnostics Society held in Congress Innsbruck, Innsbruck, Austria on the 11th May 2011. For the first time the HNODS Annual Scientific Meeting was held in association with the International Photodynamic Association (IPA) and the European Platform for Photodynamic Medicine (EPPM). The aim was to enhance the interdisciplinary aspects of optical diagnostics and other photodynamic applications. The meeting included 2 sections: oral communication sessions running in parallel to the IPA programme and poster presentation sessions combined with the IPA and EPPM posters sessions.

## Introduction

The anatomy of the head and neck is unique and complex. However, since the oral cavity and the aero-digestive tracts are easily accessible to direct examination it is feasible that disease process can be detected at an early stage. Nevertheless, in as many as 40% of patients, the disease at presentation is already at an advanced stage (T3/T4). This is unfortunate, since, the diagnosis and management of dysplastic changes or frank malignancy at an early stage would result in favourable outcome for majority of these patients [1,2]. The outlook will improve even further if simple techniques were available for surveillance and early detection of malignant transformation, and, recurrence following management. It will have a positive impact not only on improving the survival rate, but also preservation of function with low morbidity and better quality of life.

Successful management of early lesions encompasses continuous monitoring for detection of recurrence. Although the standard approach is by clinical examination with surgical biopsies, the optimum conditions for effective follow-up are not always available. The Inadequacy of documentation and imaging, relative inexperience of trainee grades, and the ever increasing clinical workload which is matched only by sparse funding are some of the factors which influence assessment for regression or progression. The implementation of "diagnostic aids" can help bridge this gap. A reliable, user-friendly and less skill-dependent technique will take away the multivariate factors which currently determine the quality of monitoring. These methods help reduce uncertainty and help guide the clinician to a diagnosis reducing waiting times and hopefully improving outcomes.

The optical diagnostic aid provides a non-invasive and real-time diagnosis. Several aids are already in use, including brush biopsy, vital staining, molecular markers, cytology and chemiluminescence [3]. However several factors limit their availability and interpretation of the results requires highly trained personnel.

### 1. What is optical diagnostics?

Optical diagnostics 'biopsy' (optical biopsy) involves the use of light of varying wavelength to examine the suspect tissue. The optical diagnostic methods are not aimed at differentiating between normal and abnormal tissue, since this can easily be achieved by direct or endoscopic visualisation. Optical diagnosis aims to provide differentiation of areas of similar clinical characteristics, i.e. dysplasia vs. carcinoma *in situ *[4-7]. Optical biopsy interrogates areas of pathology such as hyperkeratosis, inflammation, dysplasia, carcinoma *in situ *and neoplasia. Of course the technique should be minimally invasive and provide diagnosis in real time and *in vivo*. There are a number of "*in vitro" *and 'immediate "*ex vivo" *modalities in common use and they include: elastic scattering spectroscopy, deferential path-length spectroscopy, near infrared spectroscopy, Raman spectroscopy, confocal spectroscopy, fluorescence imaging, microendoscopy, and optical coherence tomography.

### 2. Elastic Scattering Spectroscopy (ESS)

Exposure of tissue to light results in a series of absorption and scattering events. The light beam undergoes single or multiple *elastic *scattering which implies that the light returns with the same energy as the incident photons. The extent of scattering effect depends on the relationship between the light wavelength and the particulate size.

A scattering event carries with it all the characteristics of the cellular components, which are called 'scattering centres.' These centres can be normal or pathological. Normal centres are nucleus, nucleolus, chromatin content and metabolic organelles. Pathological centres may be in the form of disorganized epithelial orientation and architecture, changes in morphology of epithelial surface texture and thickness, cell crowding, increased distance from sub-epithelial collagen layer, enlargement and hyperchromicity of cell nucleus, increased concentration of metabolic organelles and presence of abnormal protein packages or particles.

These cellular and sub-cellular changes are identified by using the refractive indices of the cellular components. The resultant scattered light (elastic light scattering process) is then collected and analysed by a spectrometer and a spectrum is generated [8-10].

The light emitted by cellular and sub-cellular organelles ranges from 330 nm to 850 nm, which is within the near UV and visible part of the spectrum. The ESS system is thus designed to cover a range of 300 - 900 nm and consists of a pulsed xenon arc lamp for the light source, a PC compatible spectrometer, an optical fibre (graded-index) based probe, and a laptop computer for system control and data display.

The system has a fibre-optic probe which incorporates two optical fibres, one to transmit the light into the tissue and the other for collecting scattered light. The tip of the probe is momentarily placed in direct contact with the suspected lesion and activated at the keyboard or by the foot pedal. The system takes a background measurement before firing the lamp. This is followed immediately (within-100 ms) by an ESS measurement with the pulsed lamp. The background spectrum is then subtracted from the ESS spectrum. The entire measurement processing display takes less than 1 second.

The clinical application of this emerging modality in the head and neck is still limited. Three studies carried out at the UCLH Head and Neck Centre, London, showed promising results on metastatic cervical lymph nodes (immediate *ex vivo*), cancerous bony mandible (archived, *in vitro*) and dysplastic oral lesions (*in vivo*). A sensitivity and specificity indices were 98% and 68% for lymph nodes, 87% and 80% for mandible, and 72% and 75% for dysplastic oral lesions, [8-10]. In a recent *in vivo *study (unpublished data) differing optical spectra (signatures) were obtained specifically with basal cell carcinoma, seborrhic keratosis, fibroepithelial polyp and intradermal nevi from 73 patients with suspicious head and neck skin lesions. These spectral allowed the accurate differentiation of these benign and malignant lesions with a high degree of accuracy.

Muller et al [11] used ESS to compare data from normal and abnormal tissue in the oral cavity. Using histopathology as control, the accuracy for spectroscopy for normal was 91.6% and for abnormal, 97%. An *in vivo *study was carried out at the National Medical Laser Centre, London to detect high grade dysplasia and cancer, and differentiate it from inflammation in Barrett's oesophagus. The results showed 92% sensitivity and 60% specificity in detecting high grade dysplasia or cancer. The differentiation from inflammatory lesion showed sensitivity and specificity of 79% [12].

ESS requires a close collaboration between clinicians and physicists to cleanse and normalise the obtained spectra and interpret the algorithms. Statistical support can be difficult and time consuming. Co-registration can be a problem since optical signature reports pathology changes from just 1 mm^3 ^area. Therefore, with inherent 30% shrinkage due to formalin, multiple areas need to be interrogated and then compared with the 'gold standard' histopathology. Detection of cancer in as small an area as 1 mm^3 ^would indicate a definitive treatment strategy. The drawback is lack of tumour margin detection which would entail treating much larger area than that detected positively. The advantage of the technique is the objective automated interpretation reducing user error and steepening the learning curve of the operator. It also allows good quality control.

### 3. Differential Path-length Spectroscopy (DPS)

Differential Path-length Spectroscopy is a minimally invasive technique able to determine intrinsic *in vivo *optical properties. This technology was developed at the Erasmus Medical Centre, Rotterdam. DPS is considered to be a form of elastic scattering spectroscopy but with fixed photon pathlength, fixed photon visitation depth and absolute measurement of absorbers [13]. Optical pathlength of photons is constant within reasonable range of optical properties. The signal combines information about intracellular morphology, cell biochemistry (bilirubin/betacarothene) and microvascular properties (oxygen saturation and average vessel diameter).

The system is a fibre-based diffuse reflection spectrometer with a tungsten-halogen lamp as a white light source. The first spectrometer uses a bifurcated fibre for illumination and collection. A second fibre carries diffusely reflected light to a second spectrometer. Each spectrometer records a spectrum with a slightly different wavelength scale. Subtraction of two measurements selects superficially scattered light.

DPS was used to determine the superficial optical properties of oral mucosa *in vivo *[14], bronchial tree [15] and breast tissue *in vivo *[16]. These studies showed that the malignant tissue at these sites was characterised by a significant decrease in micro-vascular oxygenation, higher blood content, significant decrease in scattering amplitude and increase in scattering slope, as compared to the normal tissue.

### 4. Near infrared spectroscopy

Near infrared (NIR) spectrum (800-2500 nm) is currently being used in medical diagnostics, pharmaceuticals and combustion research. Absorption and transmission of NIR light in biological tissue provides information about haemoglobin concentration (i.e. oxy- and deoxy-hemoglobin) [7]. Although NIR light penetrates further into tissue compared to other modalities, molecular absorption is quite small and therefore, sensitivity is not high. Since the spectra generated from the near infra-red region depend on molecular overtone and combination vibrations, complex spectra thus generated are hard to analyse.

The potential role of infrared spectroscopy in biomedical science has been proposed to distinguish different biomolecules by probing chemical bond vibrations and using these molecular and sub-molecular patterns to define and differentiate pathological from healthy samples as well as distinguishing various kinds and grades of neoplasia in human tissues [17].

### 5. Raman spectroscopy

Raman spectroscopy is used in physics and chemistry to study vibrational and other low-frequency modes in a system, enabling chemical characterisation and structure of molecules in a sample. It relies on inelastic scattering (loss or gain of energy) of monochromatic light, usually from a laser in the visible, near infrared or near ultraviolet range. The laser light interacts with molecular vibrations, resulting in the energy of the laser photons being shifted up or down. The shift in energy gives information about the photon modes in the system. Raman spectroscopy is being considered as complementary or even as an alternative technique for biopsy, pathology and clinical assays in many medical technologies [7,18].

Raman bands, due to biological constitutes, are generally overlapped (Raman effect), making it difficult to identify individual components correctly. Furthermore, due to the minimal sample preparation encountered in the clinical environment, biomedical samples tend to produce a strong fluorescent background which may completely obscure the true Raman signals.

Although Raman spectroscopy has been investigated for several decades, clinical studies in head and neck area are scant. In oncology, Raman spectroscopy is being investigated as a diagnostic tool for characterising early malignant changes. Using human tissues, researchers from University Hospital, Groningen, reported a study involving 37 human oral mucosa lesions, showing clear variations between different cell layers (keratin/epithelium versus connective tissue layer), [19]. Raman spectroscopy is a highly sensitive and specific technique for demonstration of biochemical changes in the carcinogenesis of Barrett's oesophagus. Biophotonics Research Group at the Gloucestershire Royal Hospital reported several techniques to diagnose oesophageal adenocarcinoma at an earlier stage [20].

Forty blood samples were obtained from patients with head and neck cancer and patients with respiratory illnesses, who acted as controls. Raman spectroscopy was carried out on all samples with the resulting spectra being used to build a classifier in order to distinguish between the cancer and respiratory patients' spectra. A preliminary study demonstrated the feasibility of using Raman spectroscopy in cancer screening and diagnostics of solid tumours through a peripheral blood sample [21]. Neural network analysis applied in the discrimination of human thyroid cell lines provided 95% accuracy for identification of cancerous cell lines and 92% accuracy for normal cell line [22]. Detection sensitivity for parathyroid adenomas was 95% and hyperplasia was 93% [23].

The signal generated by Raman scattering is very weak and pose a diagnostic difficulty. Preliminary studies are very encouraging. Diagnostic accuracy can be complemented by combining this technology with infra or near infrared spectroscopy.

### 6. Confocal reflectance microscopy (CRM)

Confocal reflectance microscopy (CRM) is a non-invasive imaging tool enabling "optical biopsies" of tissues at *cellular *level. CRM differs from a conventional microscopy in that it uses a point source of light, typically a laser, to illuminate a small spot within tissue. Backscattered (or reflected) light from the tissue is captured through an aperture, which matches the size of the illuminated spot placed in front of the detector. The aperture spatially filters light returning from out-of-focus planes within the tissue, imaging only the plane in focus. The gray-scale image created is an *optical *section representing *one *focal plane within the examined specimen.

This technique can supply detailed information of the epithelium 0.1-0.5 mm in depth. Since the 1980s, several investigators have shown the utility of confocal microscopy for imaging human and animal tissues *in vivo *[24]. More recently, confocal imaging has shown stronger differentiation between nuclear and dermal contrast and improved detection of tumours. By using a contrast agent, confocal images may be collected specifically from nuclear structures with very little interference from the surrounding dermis, resulting in 1000-fold improvement in nuclear-to-dermal contrast [25].

Confocal microscopy has demonstrated to date the most clinical utility in ophthalmology, where it has been used to image the cornea [26,27]. Intravenous fluorescein is used for angiography or angioscopy of the retina and iris vasculature. After injection, fluorescein binds extensively to serum albumin in the bloodstream. The unbound contrast diffuses across capillaries, entering the tissue and staining the extracellular matrix of the surface epithelium and the lamina propria for up to 30 mins [28]. Cell nuclei and mucin are not stained by fluorescein and therefore appear dark. The mucosal structures that can be identified after fluorescein administration include enterocytes, cellular infiltrate, surface epithelial cells, blood vessels, and red blood cells.

Confocal microscopy has been used for identifying *in vivo *cellular and intracellular changes in oropharyngeal epithelia. The most limiting factors for confocal microscopic investigation are the presence of severe hyper- and parakeratosis and extensive hyperplasia of the laryngeal epithelium in macroscopic leukoplakia. The failure of illumination of the tissue results from the higher refractive index of keratin in comparison with cytoplasm [29].

### 7. Fluorescence Imaging

All tissues fluoresce due to the presence of fluorescent chromophores (fluorophores) within them. The fluorescence can either occur as autofluoresence (if induced by UV light), or as a laser-induced phenomenon enhanced by either topical or systemic application of 5-aminolaevulinic acid (ALA). Fluorescence spectroscopy can detect these substances and provide characteristic spectra that reflect biochemical changes occurring within the tissue [6,7]. Commercially available fluorescence imaging equipment aims at highlighting malignant tissue, especially where it is not evident under white light in a large field of view.

An optical endoscope can be used for both illumination and detection of the tissue fluorescence. The images are captured by a frame-grabber fitted with an analogue/digital converter (ADC) and analysed and displayed on a personal computer supplied with the appropriate software. At the Ludwig Maximilian University, Munich, a great deal of work has been undertaken using autofluorescence laryngoscopy, and 5-ALA laryngoscopy. In a prospective study [30], both autofluorescence laryngoscopy, and 5-ALA laryngoscopy was undertaken in 56 patients with precancerous and cancerous lesions of the cord. They reported the following results: during autofluorescence endoscopy, normal laryngeal mucosa presented a typical green fluorescence signal in, which turned blue during 5-ALA-laryngoscopy, both imaging techniques were suitable to distinguish benign from precancerous or cancerous lesions. Fluorescence was absent during autofluorescence endoscopy for precancerous and cancerous lesions, whereas increased protoporphyrin IX fluorescence (PPIX) was noted during 5-ALA laryngoscopy. PPIX fluorescence was easily recognized in scarred vocal folds. Both autofluorescence and 5 ALA induced PPIX imaging techniques are useful in the early diagnosis of laryngeal cancer. Moreover autofluorescence can be used immediately without drug application and possible side effects. 5-ALA-induced fluorescence seems to be more suited for diagnostic examination of mucosal lesions in recurrent precancerous and cancerous lesions after surgery.

Several studies at the MD Anderson Cancer Center, Houston found differences in spectra from normal, dysplastic, and malignant oral mucosa [31,32]. At UCLH Head and Neck Centre, London a study was undertaken for fluorescence imaging with the topical application of 5-aminolevulinic as mouth rinse in 71 patients who presented with clinically suspicious oral leukoplakia. A sensitivity of 83-90% and specificity of 79-89% were obtained between normal and dysplastic lesions [33]. University Hospital Groningen researchers recorded autofluorescence spectra from 96 volunteers with no clinically observable oral lesions. Skin colour strongly affected autofluorescence intensity. Gender differences were found in blood absorption. Alcohol consumption was associated with porphyrin-like peaks. However, all differences apart from those associated with skin colour were of the same order of magnitude as standard deviations within categories [34].

### 8. Microendoscopy

Microendoscopy involves examining tissue *in situ *at a magnification from 60× to 1000×. At this magnification, details of tissues, cells and cellular ultra-structures can be also observed to detect specific patterns for pathology, i.e. inflammation, metaplasia, dysplasia, and malignancy. The Storz Hopkins II auto-clavable microendoscope is attached to a camera system linked via a video recorder with outputs to a monitor and photo-printer. The scope is available in different sizes which can be used for different anatomical sites; the dimensions of the microendoscope are 5.5 mm diameter and 23 cm long, the vital stain Methylene blue is used.

Unlike frozen section biopsy, the microendoscope provides a real time magnification at examination and can be used to guide further surgery, biopsy or simple surveillance of large areas of suspect mucosa. A recent study at Rice University, Houston evaluated microendoscopic imaging to identify neoplastic lesions in patients with Barrett's esophagus. Subjective analysis of the images by expert clinicians achieved average sensitivity and specificity of 87% and 61%, respectively [35]. The main application with the scope is that we can have a more informed assessment of the state of the *in situ *epithelial margin when excising squamous cell carcinoma. Combined with the recent advance of vital or immunologically tagged antibody targeted staining, microendoscopy will enable histopathological assessment of the surgical margin as against the standard clinically assessed margin. The extension of this technology to the management of glottic cancer will obviously have a great impact for the preservation of the voice quality with 'endoscopically assessed' clear histological margin in real time. As with all image based or image enhanced technologies there are issues with interpretation and user learning curves to ensure consistent quality control and results.

### 9. Optical Coherence Tomography

Optical coherence tomography (OCT) uses light to determine cross-sectional anatomy in turbid media such as living tissues. OCT is analogous to ultrasound imaging except that it uses light rather than sound. The high spatial resolution of OCT enables non-invasive *in vivo *"optical biopsy" and provides immediate and localized diagnostic information [36]. OCT images are formed by dividing light from an optical source into two paths, one of which is directed to the tissue sample, and the other to a reference mirror. Light reflected from the sample is recombined with light from the reference mirror and detected, forming an interference signal only when the lengths of the sample and reference paths are matched to within a short distance termed the coherence length of the light source.

OCT has been applied in the head & neck, especially in the nasopharynx, oropharynx, and larynx in an attempt to detect areas of inflammation, dysplasia and cancer. The Beckman Laser Institute and Medical Clinic, University of California Irvine, Irvine undertook clinical studies in laryngeal pathology. OCT was coupled to a surgical microscope, allowing hands-free OCT simultaneously with visualization of the vocal cords [37,38] to distinguish benign lesions from microinvasive cancer that has violated the integrity of the basement membrane (BM) [39]. This technology has yet to deliver the promise of the recognition of basement membrane to detect its invasion by malignancy.

OCT has been used at the University College Hospital, London, in 27 patients with suspicious oral lesions to assess changes in keratin, epithelial, sub-epithelial layers and identification of the basement membrane in patients. The acquired OCT data were then compared with histopathology results. This pilot study confirmed the feasibility of using OCT to identify architectural changes in pathological tissues [40]. The main issues lie around image quality and 'histological' interpretation. Further unpublished data indicates the feasibility of OCT in assessing oral and skin resection margins in clinical setting in the very near future.

### 10. The Meeting

The aims and objectives were introduced by the Chairman, Mr Colin Hopper, in his opening address. The Chairman gave an overview on optical diagnostics and discussed the challenges in the field of optical diagnostics that suffers from shortages in clinical academics as well as funding. The overall conclusion was that the Society is growing and becoming known worldwide and have a great wealth in its members of clinical academics and scientists. This society will continue to dedicate itself to helping develop optical diagnostic techniques in the head and neck and provide training networks for the exchange of information and education. **The Officers noted that the attendance to the meeting was less than expected**. However, it was very encouraging to see many senior figures from the world of optical diagnostics, from North America and many of the established universities throughout Europe.

The Programme Committee included **Colin Hopper**, University College London, United Kingdom, **Henricus J.C.M. Sterenborg**, Erasmus Medical Centre, The Netherlands, **Waseem K. Jerjes**, UCL Medical School, United Kingdom, **Adel K El-Naggar**, The University of Texas M.D. Anderson Cancer Center, USA, **Brian J.F. Wong**, University of California Irvine, USA, **Max J.H. Witjes**, University Medical Center Groningen, The Netherlands and **Tahwinder Upile**, Barnet & Chase Farm Hospitals NHS Trust, United Kingdom.

The theme was set by the first presentation by Wong B. He highlighted the experience of the Beckman Laser Institute and Medical Clinic, University of California Irvine with regards to the use of optical coherence tomography. The results from this comprehensive presentation showed that enormous success achieved in the dynamic imaging of the larynx using optical coherence tomography. One particular interesting field was monitoring of the local tissue injury and stenosis in neonates. This was followed by a series of interesting lectures by Kowalski C on longitudinal oral mucosal tissue imaging using co-localised optical coherence tomography and micro-vascular imaging and Hamdoon Z on optical coherence tomography of the tongue papillae for patients suffering from taste disorders following chemoradiotherapy and risk assessment of oral epithelium thickness using *in vivo *OCT. Witjes M discussed the use of optical coherence tomography for classification of oral mucosal lesions as well as the Netherlands experience when it comes to this technology. The 1^st ^session completed by a very interesting lecture by Ilgner J from Aachen University Hospital on OCT imaging in middle ear diagnostics.

The second session was highlighted by 3 lectures by Jerjes W on the clinical applications of elastic scattering spectroscopy in head and neck, Witjes M on single fiber reflectance measurements of cervical lymph nodes for identifying metastasis from oral carcinoma and a very interesting lecture by Cook R on micro-vascularoscopy and discussed its applications as a valuable technique for localised optical tissue assessment. The theme of the last session was on the use of optical technology to guide and monitor treatment. The lectures included discussions on the in vivo quantification of photosensitizer concentration using optical spectroscopy, non-invasive monitoring of photodynamic therapy of oral cancers by fluorescence differential path-length spectroscopy, determination of tissue optical properties in ALA-mediated head & neck PDT, monitoring photodynamic therapy using quantitative reflectance and fluorescence spectroscopic measurements, optical coherence tomography-guided photodynamic therapy for skin cancer, fluorescence spectroscopy during ALA/PpIX-mediated PDT of the oral cavity and treatment planning for interstitial photodynamic therapy of head and neck cancer.

### HNODS Elections 2011

The Nomination Committee included the following members: Christian S Betz, Alexander J MacRobert, Malini Olivo, Henricus JCM Sterenborg and Brian JF Wong. Forms to nominate members for election as Officers and Councillors has been sent to Council Members. It was decided that no elections to take place as there was only one candidate for Chairmanship.

The results for were announced at the end of HNODS Annual Meeting in May 2011. The Chairman from September 2011 will be Brian JF Wong and the Society's other Officers include Christian S Betz, Tahwinder Upile, Henricus JCM Sterenborg and Kristian Berg. The Society will be supported by 5 Councillors including Merrill A Biel, Adel K El-Naggar, Dominic J Robinson, Jennifer Rosen and Arjun G Yodh. The Immediate Past Chairman for 1 year will be Colin Hopper. The HNODS Council welcomed Dr Jennifer Rosen and Dr Arjen Amelink as new Council Members.

### 11. Discussion

Optical diagnostics provides non-invasive techniques that will allow improved surgical decision making with better appreciation by the clinician of the histopathological natural history of the disease. These novel technologies will also drive improved image and evidence based medical education with better documentation and audit, allowing informed clinical choices whilst operating. It would also facilitate targeted training and education in their usage and dissemination of its applications to virtually any discipline in medicine, surgery or dental practice.

Benefits to the patients are evident. Rather than having all suspicious areas excised for histopathological confirmation, only those areas which are optically proven to be suspicious will be biopsied. This will lead to reduced workload for the labs and there will be an increased positive yield, with significant resource implications.

Optically-directed excision may allow more complete disease treatment thus reducing the rate of recurrent or residual disease. The costs of a failed surgical margin are considerable in terms of increased morbidity and mortality with a halving of patients' survival, resulting in increased costs of adjuvant therapies and major revision surgery.

We anticipate the future publication of libraries of normative and pathological data to act as a standard clinical reference. This will enable the practitioner to use these validated technologies to help them in their day-to-day clinical work. We would predict that the practitioner would eventually be able to determine the diagnosis immediately by looking at the image on the screen. The technologies would, for a small initial outlay, provide the means to accurately diagnose a range of benign, malignant and infective conditions.

### 12. Conclusions

Optical diagnosis of the head and neck is a rapidly developing area of clinical research that can be readily translated to enhance patient treatment and overall quality of life. Much still needs to be achieved and granting organisations are solicited to pay attention to this specialty where relatively small investments may lead to enormous dividends in terms of improvements in treatments throughout the fields of medicine and surgery and dentistry.

## Competing interests

The authors declare that they have no competing interests.

## Authors' contributions

All authors have contributed to conception and design, drafting the article or revising it critically for important intellectual content and final approval of the version to be published.
